# Delayed Intracranial Hemorrhage in Patients Taking Warfarin with Head Trauma

**DOI:** 10.51894/001c.5127

**Published:** 2017-02-02

**Authors:** Mark Macedo, Jonathon Grima, Michael Yangouyian, Brian S. Kim

**Affiliations:** 1 Henry Ford Allegiance Health Emergency Department, Jackson, MI; 2 Henry Ford Allegiance Health Emergency Department, Jackson, MI; Clinical Professor, Michigan State University College of Osteopathic Medicine

**Keywords:** warfarin, minor head trauma, delayed intracranial hemorrhage

## Abstract

**CONTEXT:**

Patients presenting to the Emergency Department after a minor head injury have been shown to have a higher incidence of delayed spontaneous intracranial bleed, even with an initial negative CT. Some institutions have initiated a protocol of 24-hour observation followed by repeat head CT scan the next day. At the authors’ institution, a 24-hour observation protocol was adopted in January 2012. The authors sought to evaluate the utility of this protocol given the apparently limited amount of data to support such a practice.

**METHODS:**

A 12-month prospective observational study conducted at a community hospital evaluating a 24-month observation protocol for patients presenting after a minor head injury. During this study period, a sample of minor head injury patients were identified and followed through their hospital course. CT scan results were also compiled, and patient charts were reviewed at 30 days for any return visits or post-injury complications.

**RESULTS:**

A total of 51 patients were enrolled during the study period. Of those patients receiving a repeat head CT, none showed any evidence of a new or worsening intracranial bleed, with all patients discharged safely from the hospital. At 30 days, patients’ charts were reviewed to examine whether any patients had returned to the hospital for any reason. No sample patients returned to the hospital within the 30-day review period.

**CONCLUSION:**

Similar to the results of the retrospective chart review, no sample patients with minor head injuries showed any evidence of a subsequent delayed intracranial bleed during the 12-month study period. Our study results suggest that 24-hour observation protocols with costly repeat CT head scans may not be useful in the management of most head trauma patients. Patients may be better served to be discharged home with education including signs of symptoms of a worsening bleed (i.e., confusion, worsening headaches and not feeling right) that actually warrant a return to the hospital.

## INTRODUCTION

Since its approval for use in the 1950s, Warfarin (also known as Coumadin) has become one of the most widespread used anticoagulant medications with a fairly low side effect profile, and well understood mechanisms of absorption, metabolism, and elimination.^1^ After decades of studies concerning possible complications of systemic anticoagulation, Warfarin remains the anticoagulant by which others are generally measured.^1^ One of the more recent complications of Warfarin therapy cited in the literature is that of the delayed intracranial bleed, after even fairly minor head trauma.^2–4^ Minor head trauma has been defined as a Glasgow Coma Scale (GCS) score of 14 or 15 (possible 3 (maximum number of deficits) to 15 (no deficits) range) and a negative CT scan of the head.^2^

During the 1990s, delayed intracranial bleed after head injury in patients on Warfarin therapy was first described in case reports.^3,4^ These reports noted that minor head trauma patients on Warfarin with initially clear CT scans after were at increased risk of a spontaneous intracranial bleed, even without repeat trauma.^2^ Following this finding, the recommendation to admit all patients on Warfarin for 24-hour observation with a repeat head CT scan after admission became part of the 2002 European Federation of Neurological Society guidelines for minor head injury patients on Warfarin therapy.^4^ Complicating this issue, the exact time frame for risk of increased intracranial bleed remains unknown, with some studies suggesting such a bleed may occur days or even weeks after the initial injury.^2,5,6^

This recommendation has been adopted by some emergency departments (ED) in the United States, despite lack of definitive evidence that such policies would lead to increased detection or prevention of delayed intracranial bleeds.^2,5–7^ Widespread adaptation of this policy has led to considerable cost and time spent by physicians, patients, and hospitals in managing these patients, as well as additional radiation exposure. In this prospective observational study, the authors evaluated the utility of a 24-hour observation protocol including a repeat head CT scan.

### Purpose

This study was conducted to evaluate the efficacy and utility of admission and repeat head CT scan protocol for patients who had sustained minor head injuries after a ground level fall, presented with a normal or near-normal GCS and were on Warfarin therapy. The primary goal of the study was to evaluate the utility of a 24-hour observation protocol for potential complications from the fall that could be attributed to a delayed intracranial bleed. Most importantly, the study sought out to define the clinical importance and subsequent management of such events.

Although the phenomenon of the delayed bleeds after minor head trauma may indeed exist, the authors examined whether such an observation protocol and repeat imaging would add to, or change, the overall management of these patients. Based on the authors’ experiences, they had hypothesized that such a 24-hour observation and reimaging protocol would prove unnecessarily costly and ultimately unhelpful in the management of these patients.

## METHODS

This study was designed as a prospective observational study specifically examining those minor head trauma patients who reported to the emergency room after a ground level fall while on Warfarin therapy. The study was conducted at a community hospital in Southern Michigan with an annual ED census of roughly 100,000 visits. Before data collection, approval for this study had been obtained from the institutional review board.

### Study Sample

Physicians in the ED were asked to enroll and consent any minor head trauma patient aged 18 years or older who were on Warfarin and reported to the ED after a ground level fall. The study excluded patients younger than 18 years of age or who were not currently taking Warfarin.

### Measures and Timeline

The study was conducted from February 2014 through January 2015. On initial evaluation, the data collected included presenting GCS scores, results of head CT scans, and presenting international normalized ratio (INR) values. INR values serve as an important marker of current state of anticoagulation, allowing for verification of adherence to Warfarin medication regimen and providing a quantitative measurement of the patients’ anticoagulation status. Some studies have suggested that a higher INR increases the risk of both an immediate and delayed intracranial bleed and can be used as a marker of degree and severity of patients’ anticoagulation status.^7^

As a general hospital protocol initiated several years prior to this study, anticoagulated patients with an isolated head injury and no other reason for admission were typically admitted to the hospital’s overnight observation unit for 24-hour observation and a repeat head CT. Patients received a follow up CT the next day, with their disposition determined depending on the condition of the patient and head CT results. Patients who were not admitted to the observation unit but otherwise eligible for the study were also examined for follow-up imaging and 30-day readmission rates. These sample patients were found to possess comorbid conditions requiring admission for stabilization, were discharged home at the discretion of the attending physician, and/or signed out against medical advice.

The study hospital was a 475-bed facility without a major competing hospital within a 30-mile proximity. As such, patients enrolled in the study were evaluated for readmission to the ED or the hospital for any reason at 30 days after initial presentation. It was an assumption of the authors that if any study participants had experienced a complication or were symptomatic from a delayed intracranial hemorrhage that the most available hospital with facilities capable of managing such complications would be the study hospital.

All head CT scans that were completed were non-contrast in nature and interpreted by staff radiologists. A positive result in the initial head CT was defined as any evidence of intracranial bleed. All positive head CT results were included in the study with repeat CTs evaluated for interval change in size or severity of bleeding.

### Outcome Measures

The primary outcome measures of interest were related to progression of intracranial bleeds or new bleeds after an initial negative CT head scan. Any new intracranial findings with follow-up CT scans, whether clinically significant to patient outcomes or not, was considered a positive finding. A second outcome measured in this study was readmission to the ER or hospital for any reason within 30 days of initial head trauma.

## RESULTS

During the 12-month study period, a total of 51 adult patients were identified by the authors as being eligible for the study. The final study sample demographics consisted of 30 (59% of total) females and 21 (41%) males, with their mean age averaging 71.8 years and ranging from 47 to 98 years (see Table 1). As indicated earlier, the GCS was used to evaluate all patients on initial presentation. Using eye movement, vocalizations, and motor function, a standardized score from 1 to 15 was calculated, with a GCS of 15 indicating someone without evidence of any deficit. One sample patient presented with a GCS of 12, with the remainder of the patients either starting with a GCS of 14 or 15. For the entire sample, the mean INR level on presentation to the ER was 2.38 (SD) 1.02 (see Table 1). INR is a lab value used to measure therapeutic effectiveness of Warfarin and to help guide medication adjustments. The therapeutic goal of most patients being a value of 2, with a value of 1 being a normal level.

**Table 1:** Demographics of Sample Patients and Summary of Hospital Course

**Total Population**	51
**Median Age (range, SD)**	71 (47-98)
**Male (%)**	21 (41%)
**Presenting GCS (%)**	
15	48 (94%)
14	2 (4%)
<13	1 (2%)
INR (mean, std deviation)	2.38 ± 1.02
>2.0	23 (45%)
1.5 - 2.0	17 (33%)
<1.5	6 (12%)
**Disposition**	
Observation	21 (41%)
Admission	14 (29%)
Discharge	15 (29%)
Left AMA	1 (2%)
**Return to hospital 30 days**	0 (0%)
**Initial CT**	
Positive	2 (4%)
Negative	48 (94%)
Refused	1 (2%)
**Follow up CT**	
Negative	19
Not done	29
Positive / changed	0

**Figure 1:**
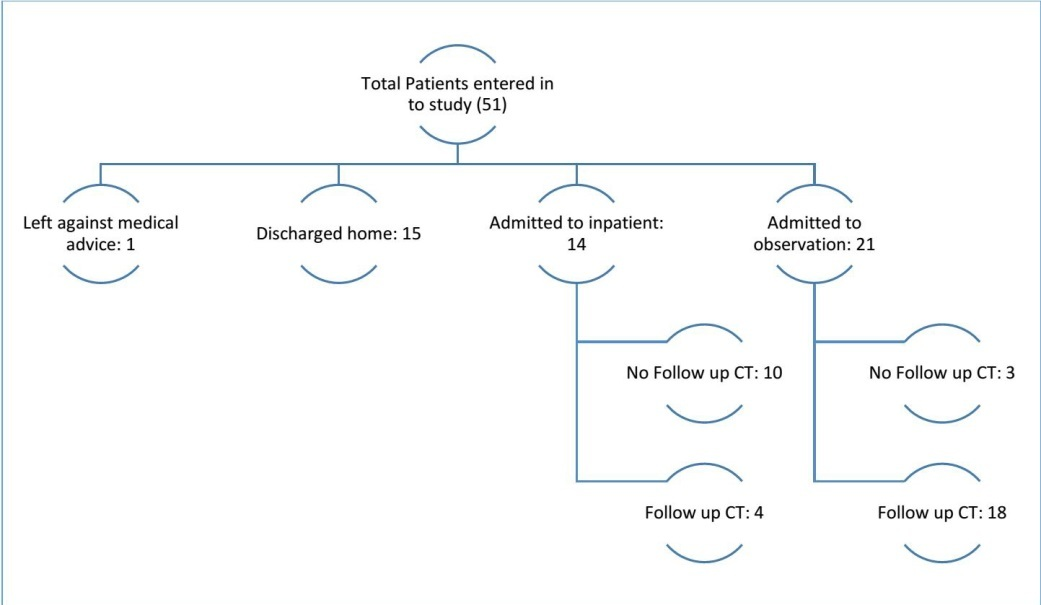
Hospital Course from Presentation in the ED through Discharge

Of the 51 sample patients, 21 (41%) were admitted for 24-hour observation, and 15 (29%) were discharged home from the ED with primary care follow up. An additional 14 (27%) patients were admitted to the hospital, and one (2%) patient left against medical advice. A total of 19 (38%) of study patients received a follow up CT the next day for evaluation of possible new onset bleed or progression of existing bleed (see Figure 1). One patient in the study received a follow up MRI in the place of a CT to evaluate for the possibility of an ischemic stroke as the cause of her fall. Of the 51 patients enrolled in the study, two (4%) had positive findings on the initial CT, and one (2%) person refused the initial CT. Finally, all of the patients who had initially enrolled in the study survived their hospital stay.

In evaluation of the 19 patients provided follow up imaging within 24 hours after initial head trauma, no patients were found to have developed a new intracranial bleed. Two of these 19 (11%) patients did have a positive finding on initial CT of intracranial hemorrhage, and follow up imaging done the next day for both of these patients failed to show any interval change (development of a new bleed) or progression of intracranial bleed. Of those 21 patients admitted for observation, six (29%) of this sample subgroup were determined to not need a follow up CT on Day Two of their stay.

**Figure 2:**
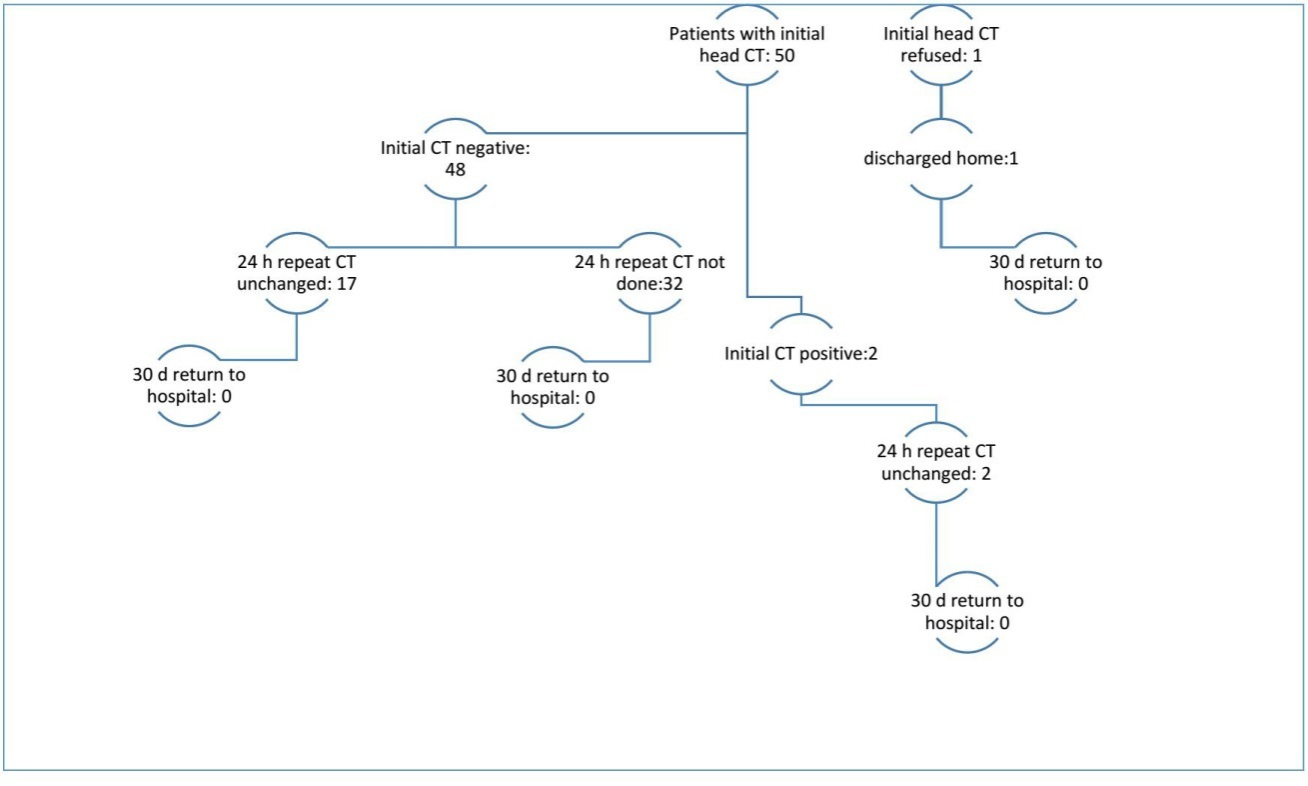
Summary of Initial Head CT Findings and Subsequent Imaging

In contrast, of those 14 admitted for inpatient services, only four (29%) of this group had any follow up imaging done to evaluate for a potential bleed based on managing provider discretion (see Table 2 and Figure 2). Presumably, those patients who were admitted were globally more unstable as a whole, yet only 29% of them were imaged on Day 2 compared to 71% of those brought in for observation. None of the enrolled patients were readmitted to our facility within 30 days of discharge.

**Table 2:** Patient Progression through Hospital Course and Subsequent Imaging Completed based on Disposition from ED

Disposition and Hospital Course	
**Observation**	21
Initial Head CT done	21 (100%)
Follow up head CT done	15 (71%)
**Admission**	14
Initial Head CT done	14 (100%)
Follow up head CT done	4 (29%)
**Discharged from ED**	16
Initial Head CT done	15 (94%)
30 day return to hospital	0 (0%)
**Discharged from hospital**	35
30 day return to hospital	0 (0%)

## DISCUSSION

These initial reports provide evidence regarding the low level of utility of a 24-hour observation and repeat head CT protocol for anticoagulated patients who have sustained minor head trauma. None of the authors’ sample patients suffered from a delayed intracranial hemorrhage. Our study results evaluated data from eligible patients presenting to one Midwestern ED for one year in total. Not one single case of delayed intracranial hemorrhage was detected during this entire analytic period. This finding calls in to question the true prevalence of delayed intracranial bleeds for minor head trauma cases and raises the question of whether or not concern over delayed post traumatic hemorrhage may be exaggerated.

It has been well documented in the literature that being on Warfarin therapy does present genuine immediate increased risk of intracranial bleed in patients with head injuries.^6,7^ Some studies suggest as many as 17% of such patients can present with an intracranial bleed ^2,6,8^ and American College of Emergency Physicians guidelines recommend an immediate initial head CT.^7^ Reviews of the literature on delayed bleed rates, however, reveal that this phenomenon is inconsistently tracked or studied.^5,7,9,10^ In fact, the results of previous studies have suggested that bleeds of this type may occur days to weeks after initial injury.^7,10^

As this and previous studies have demonstrated, delayed intracranial bleeding as a phenomenon has proven to be both unpredictable and fairly rare.^5,8,10^ The lack of any delayed bleed episodes in our sample further calls in to question the utility of a 24-hour observation and reimaging protocol. Such protocols very likely result in increased hospital costs and physician workload as well as greater unnecessary radiation exposure to patients.^6^ Until providers develop an improved evidence-based understanding of the etiology and mechanism of delayed intracranial bleeds, the benefits derived from such costly observation and reimaging protocols should be questioned.

The second part of our study involved 30-day follow up and chart evaluation to assess for readmission to the hospital due to complications from a delayed intracranial bleed. Once again, the fact that none of the study subjects returned to the hospital is telling in this case. Previous studies in this area have demonstrated that the large majority of patients showing some evidence of delayed bleeds do not have a significant enough bleed to require more than medical observation.^5–7,9,10^ It should be noted that the patients in this study each presented with a low risk mechanism and a normal or near-normal GCS. Certainly, more data needs to be gathered to better understand the nature of these delayed bleeds. Until then, however, the author’s findings match earlier and concurrent studies indicating that the chance of a massive, sudden, and life threatening hemorrhage in a patient that generally looks well with negative initial head CT findings is very low.^2,5,7–10^

### Limitations

The generalizability of our study results to other settings is limited since these findings are from a single center study site with a smaller sample. A larger multicenter study would help to validate the results of this study. Additionally, some sample patients in this study did not receive a repeat head CT scan as per the protocol being evaluated. These protocol deviations were most likely due to treating physicians’ clinical judgment that a repeat CT scan was not warranted. Although we found no evidence of any missed cases of delayed intracranial bleeds, it may also be possible that some sample patients presented to other institutions after discharge with symptoms that warranted additional evaluation and treatment.

## CONCLUSION

Despite these limitations, our study results provide additional evidence challenging the current protocol of keeping anticoagulated patients in the hospital after minor head trauma, even with an initial negative CT scan. Patients should certainly be made aware of the risks and signs of delayed intracranial bleeding, and providing them adequate follow-up care and sufficient social support. Given the uncertain nature and timeline of delayed intracranial bleeds, these results indicate that effective patient and family education may be of far greater utility than costly 24-hour observation protocols. The study findings support previous works demonstrating that ongoing observations and costly repeat imaging are unwarranted in many cases and ultimately consume valuable hospital resources.^6^

### Conflict of Interest

The authors declare no conflict of interest.
